# The PRIDE database resources in 2022: a hub for mass spectrometry-based proteomics evidences

**DOI:** 10.1093/nar/gkab1038

**Published:** 2021-11-01

**Authors:** Yasset Perez-Riverol, Jingwen Bai, Chakradhar Bandla, David García-Seisdedos, Suresh Hewapathirana, Selvakumar Kamatchinathan, Deepti J Kundu, Ananth Prakash, Anika Frericks-Zipper, Martin Eisenacher, Mathias Walzer, Shengbo Wang, Alvis Brazma, Juan Antonio Vizcaíno

**Affiliations:** European Molecular Biology Laboratory, European Bioinformatics Institute (EMBL-EBI), Wellcome Trust Genome Campus, Hinxton, Cambridge CB10 1SD, UK; European Molecular Biology Laboratory, European Bioinformatics Institute (EMBL-EBI), Wellcome Trust Genome Campus, Hinxton, Cambridge CB10 1SD, UK; European Molecular Biology Laboratory, European Bioinformatics Institute (EMBL-EBI), Wellcome Trust Genome Campus, Hinxton, Cambridge CB10 1SD, UK; European Molecular Biology Laboratory, European Bioinformatics Institute (EMBL-EBI), Wellcome Trust Genome Campus, Hinxton, Cambridge CB10 1SD, UK; European Molecular Biology Laboratory, European Bioinformatics Institute (EMBL-EBI), Wellcome Trust Genome Campus, Hinxton, Cambridge CB10 1SD, UK; European Molecular Biology Laboratory, European Bioinformatics Institute (EMBL-EBI), Wellcome Trust Genome Campus, Hinxton, Cambridge CB10 1SD, UK; European Molecular Biology Laboratory, European Bioinformatics Institute (EMBL-EBI), Wellcome Trust Genome Campus, Hinxton, Cambridge CB10 1SD, UK; European Molecular Biology Laboratory, European Bioinformatics Institute (EMBL-EBI), Wellcome Trust Genome Campus, Hinxton, Cambridge CB10 1SD, UK; Ruhr University Bochum, Medical Faculty, Medizinisches Proteom-Center, D-44801 Bochum, Germany; Ruhr University Bochum, Center for Protein Diagnostics (PRODI), Medical Proteome Analysis, 44801 Bochum, Germany; Ruhr University Bochum, Medical Faculty, Medizinisches Proteom-Center, D-44801 Bochum, Germany; Ruhr University Bochum, Center for Protein Diagnostics (PRODI), Medical Proteome Analysis, 44801 Bochum, Germany; European Molecular Biology Laboratory, European Bioinformatics Institute (EMBL-EBI), Wellcome Trust Genome Campus, Hinxton, Cambridge CB10 1SD, UK; European Molecular Biology Laboratory, European Bioinformatics Institute (EMBL-EBI), Wellcome Trust Genome Campus, Hinxton, Cambridge CB10 1SD, UK; European Molecular Biology Laboratory, European Bioinformatics Institute (EMBL-EBI), Wellcome Trust Genome Campus, Hinxton, Cambridge CB10 1SD, UK; European Molecular Biology Laboratory, European Bioinformatics Institute (EMBL-EBI), Wellcome Trust Genome Campus, Hinxton, Cambridge CB10 1SD, UK

## Abstract

The PRoteomics IDEntifications (PRIDE) database (https://www.ebi.ac.uk/pride/) is the world's largest data repository of mass spectrometry-based proteomics data. PRIDE is one of the founding members of the global ProteomeXchange (PX) consortium and an ELIXIR core data resource. In this manuscript, we summarize the developments in PRIDE resources and related tools since the previous update manuscript was published in *Nucleic Acids Research* in 2019. The number of submitted datasets to PRIDE Archive (the archival component of PRIDE) has reached on average around 500 datasets per month during 2021. In addition to continuous improvements in PRIDE Archive data pipelines and infrastructure, the PRIDE Spectra Archive has been developed to provide direct access to the submitted mass spectra using Universal Spectrum Identifiers. As a key point, the file format MAGE-TAB for proteomics has been developed to enable the improvement of sample metadata annotation. Additionally, the resource PRIDE Peptidome provides access to aggregated peptide/protein evidences across PRIDE Archive. Furthermore, we will describe how PRIDE has increased its efforts to reuse and disseminate high-quality proteomics data into other added-value resources such as UniProt, Ensembl and Expression Atlas.

## INTRODUCTION

Data sharing in the public domain has become the standard for proteomics researchers. The growth in recent years has been very remarkable and as a result, the number of proteomics datasets deposited every year in open public repositories is now comparable to transcriptomics (1). Since 2004, the PRoteomics IDEntifications (PRIDE) database (https://www.ebi.ac.uk/pride/) at the European Bioinformatics Institute (EMBL-EBI, Hinxton, Cambridge, UK) has enabled public data deposition of mass spectrometry (MS)-based proteomics data, providing access to the experimental data described in scientific publications (2). Since then, and especially in recent years, PRIDE Archive (the archival component of PRIDE) has become the largest repository for proteomics data sharing worldwide (2,3).

PRIDE stores datasets coming from all proteomics experimental approaches, with a focus on discovery-driven techniques such data dependent acquisition (DDA) and data independent acquisition (DIA) bottom-up proteomics, but also top-down proteomics and MS imaging, among others. For each dataset submitted to PRIDE Archive (the archival component of PRIDE), the MS raw files (output files from the mass spectrometers) and the processed results (at least peptide/protein identification results, quantification information is optional) must be provided. In addition, each dataset in PRIDE Archive can contain peptide/protein quantitation result files, the mass spectra as peak list files, the searched protein sequence databases or spectral libraries, programming scripts, and any other technical and/or biological metadata provided by the data submitters (4). The PRIDE team has led within the Proteomics Standards Initiative (PSI) organization, the creation and implementation of multiple standard open file formats such as mzTab (5), mzIdentML (6) and mzML (7) to store, process and visualize the proteomics data deposited.

The stand-alone ProteomeXchange (PX) Submission tool (8) allows the researchers to perform the data submissions to PRIDE Archive, while PRIDE Inspector (9) enables users to review the dataset before, during, and after has been deposited in the resource. After the submission is completed, different pipelines perform the validation and quality assessment of the reported results and store the data into multiple databases for enabling data access and visualization in the PRIDE Archive web interface (https://www.ebi.ac.uk/pride/archive) and also programmatically via the PRIDE Application Programming Interface (API, https://www.ebi.ac.uk/pride/ws/archive/v2/). In recent years, PRIDE Archive has been moving its visualization components from desktop-based applications (e.g., PRIDE Inspector) to Restful APIs and web-based interfaces. All submitted files are available to download via FTP or the Aspera file transfer protocol.

PRIDE resources have two main missions for the proteomics community: (i) support data deposition and quality assessment of submitted proteomics experiments, to help reproducible research; and (ii) promote and facilitate the reuse of public proteomics data, and disseminate high-quality proteomics evidences into added-value resources, including Ensembl (10), UniProt (11) and Expression Atlas (12).

The PRIDE database was one of the founders of the PX consortium in 2011 (3,8). PX defines the guidelines for data submission and dissemination of public proteomics data worldwide. As of 2021, the resources PeptideAtlas (13), including its related resource PASSEL (PeptideAtlas SRM Experiment Library) (14), MassIVE (15), jPOST (16), iProX (17) and Panorama Public (18) are the active members of the consortium. PX coordinates the release of accession numbers for every submitted dataset and a set of services for providing unified access to publicly available datasets (http://proteomecentral.proteomexchange.org/cgi/GetDataset), including specific data types such as mass spectra, using Universal Spectrum Identifiers (19) (http://proteomecentral.proteomexchange.org/usi/). Additionally, in 2017, PRIDE became an ELIXIR (http://www.elixir-europe.org) core data resource (20) and ELIXIR deposition database, recognizing its key role in the life sciences.

In this manuscript, we will summarize the main PRIDE-related developments in the last three years, since the previous *Nucleic Acids Research* (NAR) database update manuscript was published (2). We will discuss PRIDE Archive first but will also provide updated information about the PRIDE-related tools and other ongoing activities including the updates in the PRIDE Spectra Archive and PRIDE Peptidome. Additionally, we will also report about the work performed to disseminate and integrate proteomics data in other EMBL-EBI resources.

## CURRENT STATUS OF THE PRIDE ECOSYSTEM: RESOURCES AND TOOLS

**Figure 1.**
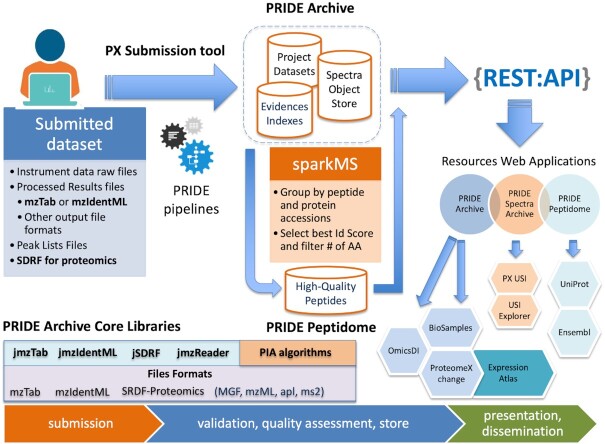
Schema of the PRIDE resources ecosystem. PRIDE Archive users must provide the raw files, the processed results files, and metadata about every given dataset. Standard file formats (for processed result files) can be provided for 'Complete' submissions. A group of open-source libraries is used by the PX Submission tool, and the PRIDE pipelines to validate, assess the quality of the reported peptides and proteins, and store the information (metadata, peptides/proteins and spectra) into multiple databases. The PRIDE Peptidome resource selects high-quality peptides across all the datasets in PRIDE Archive. All the data from PRIDE Archive and PRIDE Peptidome is served to external users such as Ensembl and UniProt through the PRIDE API and PRIDE web interface. Additionally, proteomics quantitative datasets are reanalyzed and integrated into Expression Atlas.

The PRIDE database ecosystem (https://www.ebi.ac.uk/pride/) is composed of a comprehensive set of libraries, desktop tools, databases, large-scale pipelines, Restful APIs and web applications (Figure 1). A set of open-source Java libraries including jmzTab (21), jmzIdentML (22), ms-data-core-api (23) and the protein inference algorithms toolkit (PIA) (24,25) supported and maintained by the PRIDE team, allows to read, validate, process, and store proteomics data encoded in PSI open file formats. PRIDE Archive pipelines (2) perform a set of validation and quality checks to make sure the deposited files are semantically valid, and that the metadata provided during the submission is correct, in addition to moving the submitted datasets into the EMBL-EBI production filesystem.

When a given dataset is made public, a group of post-submission pipelines parses the peptides and proteins identified in the dataset—if the dataset is a ‘complete’ submission (4)—and index them into Apache Solr and MongoDB-based infrastructure enabling to search datasets by the identified peptides and proteins. The PRIDE Spectra Archive and PRIDE Peptidome provide access to the mass spectra identified in the PRIDE Archive and to a condensed view of high-quality identified peptides across PRIDE Archive datasets, respectively. All data from PRIDE Archive and related resources are served through the PRIDE Restful API and the web application.

### Data submission

The PRIDE Archive guidelines for data submission including the required data files and metadata have not changed substantially in recent years, in parallel to PX requirements. Previous publications (2,4) explain in detail the main formats supported, the type of submissions (‘complete’ or ‘partial’), and the required metadata for each dataset. Complete submissions are those where the processed results are submitted in the PSI standard file formats mzIdentML or mzTab. A web tutorial explaining the process of submission is available at https://www.ebi.ac.uk/training/online/courses/pride-quick-tour/, explaining the main steps for data submission.

In 2019, complete submissions containing quantitative information based on the PRIDE XML file format were discontinued and replaced by mzTab-based complete submissions. mzTab (5) is a PSI tab-delimited format that supports the representation of not only identification results but also quantitative results and post-translational modification (PTM) localization information. Since 2019, Mascot (26), MaxQuant (27) and OpenMS (28) can export the resulting identification/quantification results into mzTab. Since 2020, overall, 240 and 30 dataset submissions have been performed using mzTab generated from Mascot and MaxQuant, respectively. Recently, the MaxQuant and PRIDE teams worked together to enable the novel tool MaxDIA (29) to export results from DIA approaches to mzTab.

Minor improvements have been done to the PX Submission tool including performance improvements in the OLS Dialog (30) component, which allows searching for ontology/controlled vocabulary terms in the Ontology Lookup Service (https://www.ebi.ac.uk/ols/index). As a key point, file checksums are now computed during the submission and validated by the PRIDE pipelines to ensure the integrity of the submitted files. Two additional improvements have been implemented as part of the submission process: (i) add information about datasets license; and (ii) submission of sample metadata and experimental design information using the newly developed file format MAGE-TAB for proteomics.

### Datasets licenses

Licenses for datasets stored in PX resources had not been originally defined or agreed upon (3). In 2020, PX partners decided to move towards a default Creative Commons CC0 license as a minimum level for each dataset, making it available globally datasets without any restrictions. PRIDE used to follow the EMBL-EBI ‘Terms of use’ (https://www.ebi.ac.uk/about/terms-of-use). The CC0 license can only be ensured for prospective newly submitted datasets since 2020. It is expected that for PRIDE, a CC0 license will be the default one in the foreseeable future, in parallel to the policy in other EMBL-EBI resources.

### MAGE-TAB for proteomics: improving sample metadata and experimental design

**Figure 2.**
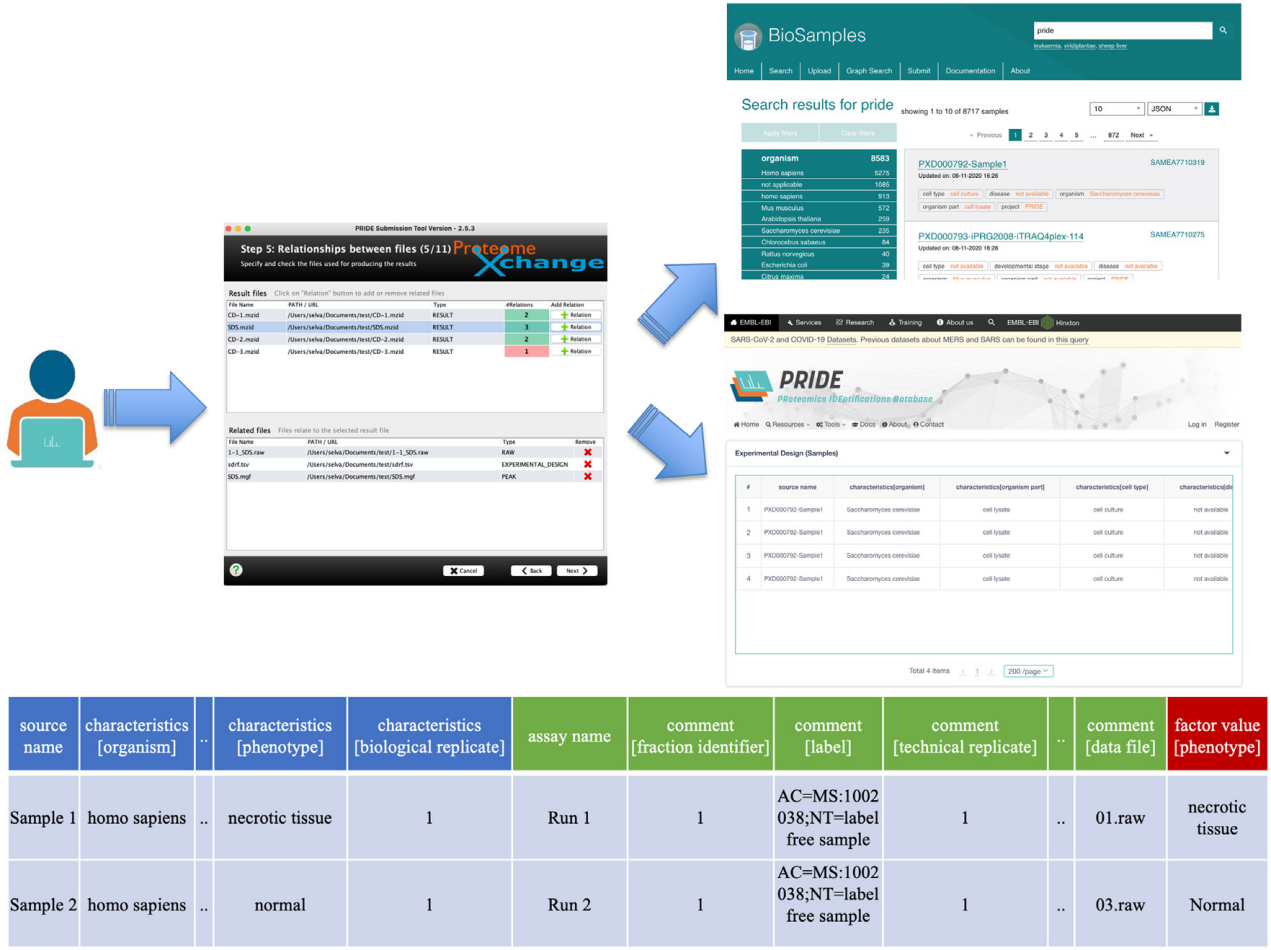
PRIDE Archive users can now provide SDRF-Proteomics files to represent the experimental design and the relationship between the samples analyzed and the instrument raw files. The samples included in the SDRF-Proteomics files are submitted to BioSamples getting each of them a unique accession number. In addition, the PRIDE web interface represents the information contained in SDRF-Proteomics files in an ‘Experimental Design’ table, including all samples and data files.

For every submitted dataset to PRIDE Archive, general metadata about the study must be provided including the title, submitters’ details, dataset description, sample and data protocols, instrument, and the associated publication once it is published (2,4,8). It has been highlighted multiple times (31–33) how the lack of appropriate metadata at the sample level, including the experimental design (e.g. samples treatment, fractionation steps, etc.), prevents a more streamlined reuse of the available data, especially in the case of reanalyses of quantitative proteomics datasets. The MAGE-TAB for proteomics (34), an extension of the format original MAGE-TAB format used in transcriptomics (35), has been recently proposed to capture the sample metadata, and the experimental design for proteomics experiments (Figure 2).

MAGE-TAB for proteomics has two main components: the Investigation Description Format (IDF) and the Sample and Data Relationship Format (SDRF). The IDF contains the general description of the study which is the same information annotated with the PX Submission tool. Then users do not need to provide it upon submission. The SDRF-Proteomics format includes the representation of the experimental design, and the relationship between the samples analyzed in the experiment and the MS data files (raw files). The SDRF-Proteomics is a tab-delimited format where each column is a property of the sample or the data file. Each row corresponds to the relation between a sample and a data file, and each cell is the value of the property for the sample or the data file (34) (https://github.com/bigbio/proteomics-metadata-standard).

SDRF-Proteomics files can now be added manually by the user and selecting the ‘EXPERIMENTAL DESIGN’ as the file type during the submission. Once the data arrives at PRIDE, a BioSample database accession is requested for each sample and added into the BioSample resource (36) (e.g. https://www.ebi.ac.uk/biosamples/samples/SAMEA7710319) via the PRIDE Archive pipelines. In addition, the corresponding experimental design table (e.g. - https://www.ebi.ac.uk/pride/archive/projects/PXD000792) (Figure 2) can be accessed through the PRIDE Archive web interface. As of September 2021, more than 130 public datasets have been re-annotated by third parties (33) and the resulting information is available via PRIDE Archive (https://www.ebi.ac.uk/pride/archive?keyword=sdrf.tsv).

### PRIDE Archive web interface and Restful API: accessing proteomics evidences

The PRIDE Restful API (https://www.ebi.ac.uk/pride/ws/archive/v2/) can be used to query and access all the data in PRIDE resources. By using the API it is possible, for example, to query and find datasets by their date of publication, the proteins that have been identified, or the name of a data file within the study (e.g., https://www.ebi.ac.uk/pride/ws/archive/v2/search/projects?keyword=Subject1_FACS145_B_C10). A powerful query language allows users to combine multiple keywords (properties of the project) into an SQL-based query to search datasets. A Python package and tool (https://github.com/PRIDE-Archive/pridepy) have been developed to programmatically interact with the PRIDE Archive Restful API. The package provides a data model for all the data structures provided by the API but also includes functionality that enables to query each endpoint in the API (see https://github.com/PRIDE-Archive/pridepy#examples).

**Figure 3.**
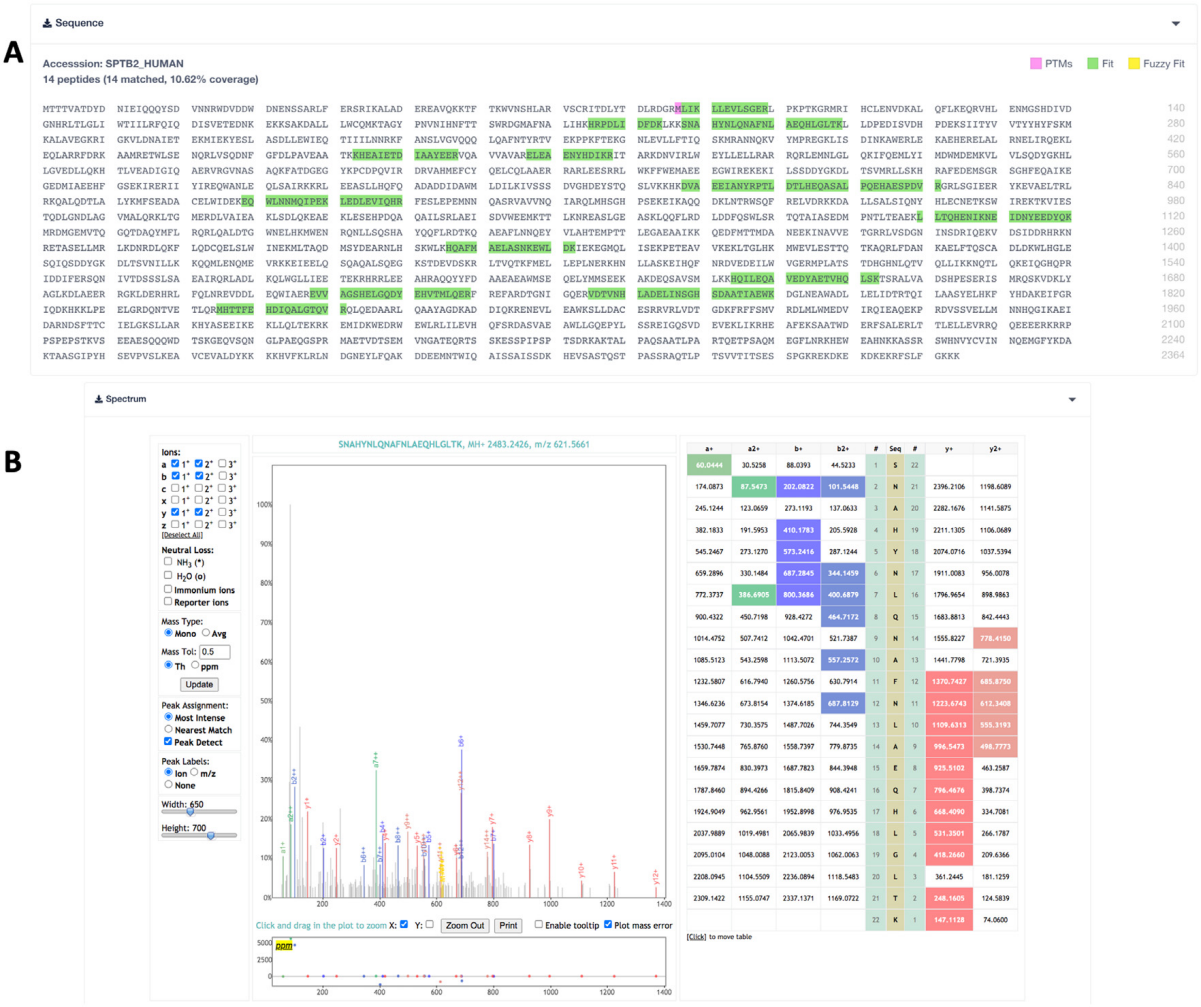
The PRIDE web interface provides functionality to assess the quality of each Complete submission, including components to: (**A**) visualize the sequence coverage of a particular protein; and (**B**) visualize the spectrum used to identify a given peptide.

The PRIDE Archive web interface provides visualization components that allow to search, find and inspect all the dataset information. A large number of the features from PRIDE Inspector have been moved into the PRIDE web, enabling the inspection of the peptide/protein evidences and the spectra identified in each complete submission (Figure 3). In the results exploration viewer, users can explore the identification results, including the protein coverage in the identified proteins and the mass spectra that are part of each PSM (Peptide Spectrum Match) (Figure 3, https://www.ebi.ac.uk/pride/archive/projects/PXD008613/results?reportedAccession=SPTB2_HUMAN&assayAccession=83415). It is important to highlight that these features are only available for complete submissions.

### PRIDE Spectra Archive: accessing and visualizing all spectra for complete submissions

The public availability and direct access to mass spectra data create the opportunity for scientists to directly assess whether, e.g., a novel peptide evidence, PTM, or amino acid variant (SAAV) are supported by a good-quality and well-annotated mass spectrum (19,37). PSI and PX partners have recently created a novel mechanism to uniquely resolve each mass spectrum in public proteomics resources. The Universal Spectrum Identifier (USI) enables greater transparency of spectral evidence making it more ‘FAIR’ (Findable, Accessible, Interoperable, and Reusable), with more than 1 billion USI identifications from over 3 billion spectra already available through PX repositories (19).

The PRIDE Spectra Archive (https://www.ebi.ac.uk/pride/archive/spectra) provides access to over 540 million PSMs (as of September 2021) originally submitted to PRIDE Archive. Users can search by peptide sequences and USIs, enabling them to find specific PSMs from complete submitted datasets. A list of PSMs is shown after the search, including peptide sequences, PTMs, search engine scores, charges, and two additional columns that highlight whether the PSM has passed or not the original analysis threshold and PRIDE internal pipelines thresholds—for example, PSM false discovery rate (FDR) <0.1 computed using the PIA algorithm (24,25). The accession column in the result table provides a direct link to the project result page, where users can check all the results for a given dataset.

### PRIDE Peptidome: a condensed view of peptide evidences across PRIDE Archive

PRIDE Peptidome (https://www.ebi.ac.uk/pride/peptidome/) is a resource that groups all PSMs by peptide sequence and the corresponding protein accession. Until recently, the grouping was performed using a spectrum clustering approach (38). However, this approach presented major challenges because each spectrum needed to be compared between each other, prompting performance challenges, due to the continuous and remarkable growth in the amount of submitted data. Although spectrum clustering algorithms have been recently improved using deep-learning models to avoid all the comparisons between all the spectra in the data (39,40), applications of these novel algorithms in large-scale data repositories have not yet been implemented.

Instead of spectrum clustering, a novel platform and algorithm (https://github.com/bigbio/sparkms) have been used to select the best-peptide evidence for each peptide and protein combination. The best peptide is selected based on two rules: (i) the peptide passes the peptide FDR threshold for the assay; and (ii) the peptide sequence is longer than seven amino acids. The sparkMS (https://github.com/bigbio/sparkms) used Spark (https://spark.apache.org/) and PySpark to group millions of PSMs in less than 6 hours, which enabled the data analysis of such a large-scale amount of data.

The PRIDE Peptidome web interface enables users to search by peptide sequence and protein accession numbers (e.g. https://www.ebi.ac.uk/pride/peptidome/peptidesearch?keyword=SPTB2_HUMAN). The search table shows the sequence for each peptide, protein accession, the number of PSMs across PRIDE Archive, the number of datasets where this peptide has been identified and the best posterior error probability (PEP), as computed by PIA (25). When a given peptide-protein combination is selected, the peptide viewer shows the sequence, the spectrum that justifies the best scored PSM, the list of all PTMs identified, and the corresponding tissues and diseases where the peptide was identified (e.g. https://www.ebi.ac.uk/pride/peptidome/peptidedetails?keyword=DASVAEAWLLGQEPYLSSR&proteinAccession=SPTB2_HUMAN).

## PRIDE ARCHIVE SUBMISSION STATISTICS

**Figure 4.**
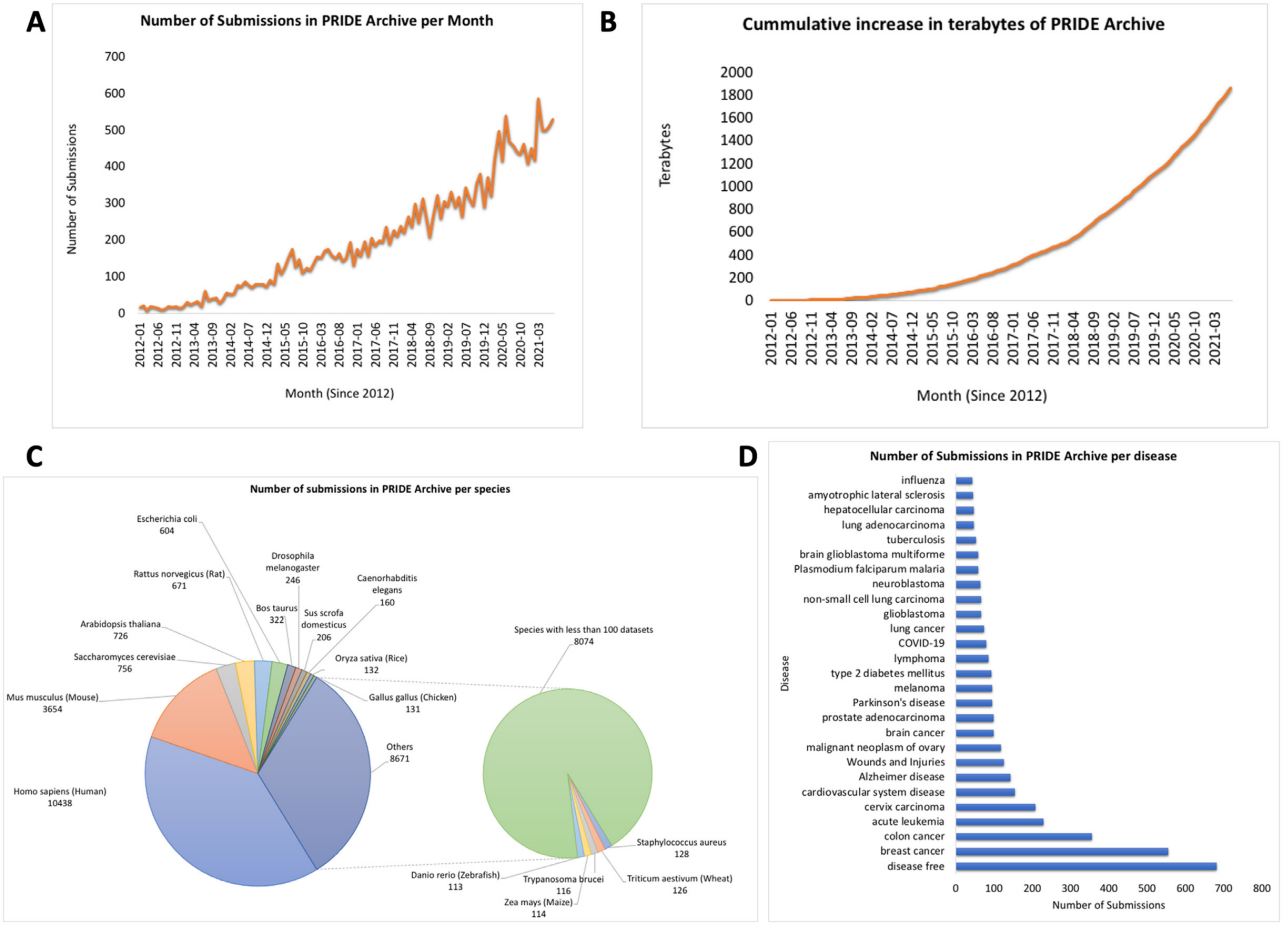
(**A**) Number of submitted datasets to PRIDE Archive per month (from the beginning of PX in 2012 till August 2021); (**B**) cumulative size of PRIDE Archive data since 2012; (**C**) number of submitted datasets per species or taxonomy identifier (as of August 2021). All species that had less than 100 datasets are grouped in one category; (**D**) distribution of the number of submitted datasets to PRIDE Archive per annotated disease.

As of 1 August 2021, PRIDE Archive stored 23 168 datasets—compared to the 10 100 datasets available on August 2018 (2)—, which means that 56.4% of the data in PRIDE Archive has been submitted in the last 3 years. Figure 4 shows the distribution of submissions by month, species, and disease in PRIDE Archive since 2012, and the cumulative size of PRIDE Archive data in terabytes.

In 2019, PRIDE Archive received 314 datasets per month on average, 436 during 2020, and so far in 2021, this number has grown to 499 datasets on average (Figure 4A), which affirms the increasing huge demand and growing prominence of PRIDE. At the time of writing, PRIDE hosts ∼83% of all PX datasets, coming from >8 000 research groups, from 66 countries. The number of submitted datasets that are now publicly available is currently 64%, reflecting an improvement of around 8% when compared with 2019. With this aim in mind, the team has developed multiple mechanisms to detect datasets already published that have not been reported to PRIDE by the original submitters. As a concrete example, submitters can report via the PRIDE web interface datasets that have already a corresponding manuscript published, if the dataset is still private. The size of PRIDE Archive data has doubled from 2019 to 2021 (Figure 4B). As a result, PRIDE Archive is the third-largest omics Archive at EMBL-EBI only exceeded by the genomics resources ENA (European Nucleotide Archive) and EGA (European Genome-phenome Archive) (41).

As of September 2021, the majority of data in PRIDE Archive (including both public and private datasets) are human datasets (including cell lines) (39.1%), followed by mouse (13.7%), *Saccharomyces cerevisiae* (2.8%), *Arabidopsis thaliana* (2.7%), *Rattus norvegicus* (2.5%) and *Escherichia coli* (2.3%). Whereas most of the datasets come from model organisms, overall, datasets coming from >3 224 different taxonomy identifiers are stored in PRIDE Archive (Supplementary File S1).

The number of submitted datasets split by tissues and diseases are more heterogeneous (Figure 4C and D), being ‘cell-culture (non-specific tissue)’, and ‘disease-free (healthy/normal samples)’ the most predominant annotations. Altogether, cancer is the most studied disease followed by Alzheimer's and Parkinson's disease. Importantly, as of September 2021, more than 180 COVID-19 related datasets have been submitted to PRIDE Archive. These datasets, once they become publicly available, are integrated into the EMBL-EBI resource COVID-19 Data Portal (https://www.covid19dataportal.org/), enabling researchers to access all public data at EMBL-EBI resources in a unified interface (42).

## PRIDE ARCHIVE AS A HUB OF MS EVIDENCES

**Figure 5.**
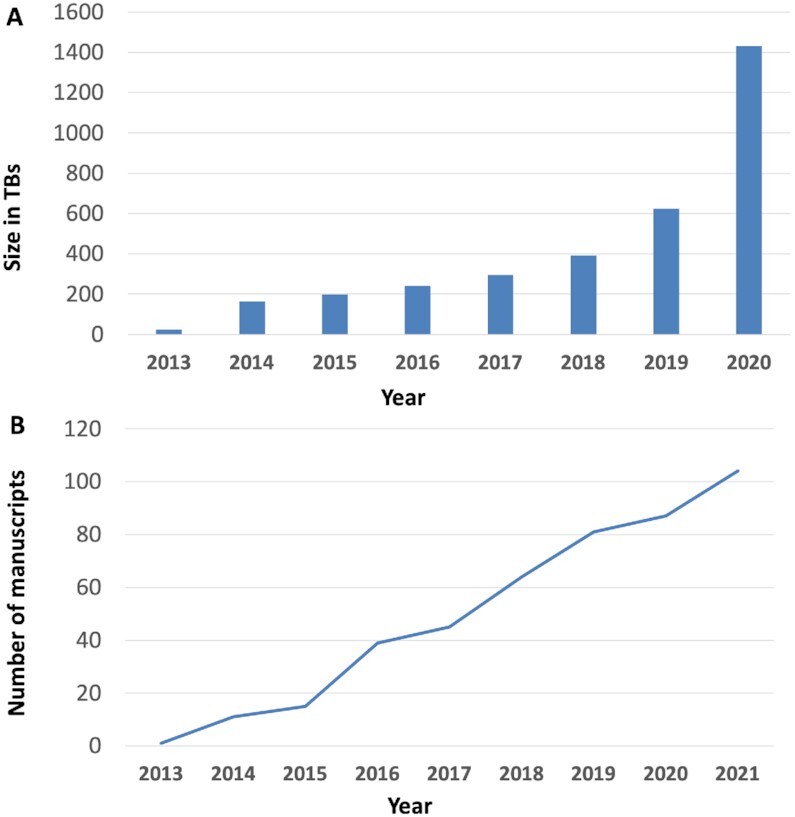
(**A**) Volumes of PRIDE Archive data downloads per year, from 2013 to 2020. (**B**) Number of manuscripts (including pre-prints) per year (2013–2021), where datasets from PRIDE Archive are reused. The figures from 2021 are estimated at the end of the year, according to the existing data at the end of September. It should be noted that the figures represent an underestimation since they only include those manuscripts that could be tracked successfully.

Proteomics researchers are increasingly reusing public data from PRIDE (and other PX resources) for a broad range of purposes. For instance, recent resources that have been started by reusing mostly PRIDE public datasets include OpenProt (43), MatrisomeDB (44), Scop3P (45) and ProteomeHD (46). Additionally, as just one among many examples of high-profile data reuse, PRIDE datasets are routinely reanalyzed in the context of the Human Proteome Project (47). Figure 5A shows the increase in volumes of data downloaded from PRIDE Archive since 2013. Recently, PRIDE has started to track the reuse of public PRIDE datasets in publications. This information (if applicable) is available in the dataset web page when clicking on the term ‘Dataset reuses’. Figure 5B shows the increase in manuscripts (including pre-prints) published per year, where PRIDE datasets are reused.

Rather than in the creation of new resources, for sustainability reasons, our focus in-house has been put in disseminating and integrating PRIDE proteomics data into added-value EMBL-EBI resources such as UniProt (11), Ensembl (10), and Expression Atlas (12). Additionally, we have just started in the first steps of the work required to disseminate and integrate metaproteomics data into MGnify (48), an EMBL-EBI resource for the analysis, archiving, and browsing of metagenomic and metatranscriptomic data. The dissemination of public proteomics data into different resources has different goals depending on each specific resource but can be grouped in three main categories: (i) provide aggregated peptide/protein evidences as originally submitted to PRIDE Archive, in the case of UniProt and Ensembl; (ii) provide peptide/protein evidences, variant sequences and PTM information from reanalyzed datasets to UniProt, Ensembl and in the near future, to MGNify. In this case, an open analysis pipeline is used, including well-defined quality control metrics; and (iii) provide quantitative protein expression information into Expression Atlas, using data coming from reanalyzed datasets.

### In-house data reuse: proteogenomics reanalysis integration with Ensembl

Since 2019, PRIDE has started to provide peptide evidences to Ensembl using the ‘TrackHub’ registry (2). More than 4 million canonical peptide sequences, coming from 184 PRIDE public datasets, have been disseminated into Ensembl ‘TrackHubs’ which are available at https://ftp.pride.ebi.ac.uk/pride/data/proteogenomics/latest/archive/.

Some obvious benefits of integrating genomics and proteomics data in genome browsers include linking somatic variants and MS evidences and/or gene sequences and PTMs. Recently, we developed a group of tools and workflows to enable large-scale reanalysis of public proteomics data to identify non-canonical peptides (49). Using custom proteogenomics databases created with pgdb (https://github.com/nf-core/pgdb) and the pypgatk (https://github.com/bigbio/py-pgatk) we have managed to identify 43 501 non-canonical peptides and 786 variant peptide sequences in four public datasets.

### In-house data reuse: data dissemination into UniProt

Aggregated high-quality evidences (as submitted to PRIDE Archive) are linked to UniProt enabling users to check whether one particular protein has been seen detected in PRIDE Archive. As part of an ongoing effort, we are currently aiming to link all peptide evidences from PRIDE Peptidome to populate the UniProt ProtVista viewer (50).

Additionally, we are currently working in the development of infrastructure to reanalyse in a reliable manner, store, visualize and disseminate PTM data (starting with phosphorylation) from PRIDE into UniProt. This is taking place in the context of the ‘PTMeXchange’ project, in collaboration with the PeptideAtlas team and the University of Liverpool. Previously to this more systematic effort, we reanalysed 112 human phospho-enriched datasets, generated from 104 different human cell types or tissues (51). Using a machine learning approach, some of the generated information from the reanalysis together with other sequence features were used to create a single functional score for human phosphosites.

### In-house data reuse: integration of quantitative analyses in Expression Atlas

More than 65 quantitative datasets have been annotated, reanalysed and the corresponding results have already or are being integrated into Expression Atlas at the moment of writing. Most of them are DDA label-free datasets, involving cell lines and tumor samples (52), and baseline tissue datasets coming from human, mouse and rat samples. MaxQuant was used as the analysis software in all cases. Additionally, ten SWATH-MS DIA datasets coming mainly from cell line and human tumor samples have also already been re-analysed and integrated into Expression Atlas. In this case, an in-house open analysis pipeline based on OpenSWATH (https://github.com/PRIDE-reanalysis/DIA-reanalysis) was developed and used for the re-analysis (53). These datasets constituted a pilot project to study the feasibility of performing a systematic reanalysis and integration of DIA datasets. Expression Atlas users can now access more comprehensively proteomics expression information in the same interface as gene expression, providing an effective manner of integrating the results of transcriptomics and proteomics experiments.

## DISCUSSION AND FUTURE PLANS

Data deposition and dissemination have changed the proteomics community since the creation of PX almost 10 years ago. Most of the proteomics journals require nowadays the authors to deposit their data in a PX resource, which has enabled a better reproducibility and traceability of the claims reported in a given manuscript. The proteomics community is now widely embracing open data policies, an opposite scenario to the situation just a few years ago. At the same time, public proteomics data are being increasingly reused with multiple applications (1). We next outline some of the main working areas for PRIDE in the near future.

First of all, PRIDE is raising the bar of metadata annotation for all submitted datasets. MAGE-TAB for proteomics has been created with the aim that every submitted dataset provides information about the sample and the experimental design. The improvement in the annotation is also required to facilitate further data reuse for third parties. We expect that, gradually, the SDRF-Proteomics component will be made required for every dataset submission, after the community understands and get a full idea of the file format and of the mandatory information that needs to be provided. Multiple materials (https://github.com/bigbio/proteomics-metadata-standard/wiki), including examples and video tutorials, have been made available to better understand the file format and how it can be submitted to PRIDE Archive.

With the growing importance of clinical proteomics, i.e. in the context of multi-omics studies, another important area is the management of clinical sensitive human proteomics data. Ethical issues in proteomics are starting to be discussed and becoming increasingly relevant. A community-driven white paper on the topic has been recently published describing the current state-of-the-art (54). Addressing ethical issues for genomics and transcriptomics data led to processes to control who may access the data, so-called ‘controlled access’. Resources supporting the storage and dissemination of controlled access DNA/RNA sequencing datasets include the EGA and others internationally such as dbGAP (USA) and the Japanese Genotype-phenotype Archive. At present, all data in PRIDE (and in all PX resources) is fully open. Therefore, there is an increasing number of clinical sensitive human datasets that cannot be made available *via* PRIDE due to ethical-related issues (55). To address this problem, we will be working in developing a tailored infrastructure for sensitive human proteomics data, and in all the related policies. Additionally, in the context of data archiving activities, we plan to improve the support for cross-linking data - as outlined here (56) - and to provide better data integration for structural proteomics datasets between PRIDE Archive and the Protein Data Bank (PDB).

As shown above, we are already working on developing open and reproducible data analysis pipelines for different flavours of proteomics workflows (e.g., DDA, DIA, proteogenomics) (49,53,57). The main rationale is to make possible the use of that software in cloud infrastructures so that in the future the pipelines can be used by the community in the cloud using software container technologies (58). In addition, we aim to increasingly perform in-house data reuse (including data re-analysis) and disseminate high-quality proteomics data from PRIDE into the already mentioned added-value resources (Ensembl, UniProt, Expression Atlas, and MGnify in the near future). In this context, we will also work in improving the PRIDE Archive infrastructure to store dataset reanalyses appropriately, linking them to the relevant resources. One aim is to further develop data dissemination and integration practices also involving resources outside of EMBL-EBI.

To finalize, we invite interested parties in PRIDE-related developments to follow the PRIDE Twitter account (@pride_ebi). For regular announcements of all the new publicly available datasets, users can follow the PX Twitter account (@proteomexchange).

## Supplementary Material

gkab1038_Supplemental_Files

## References

[1] Perez-Riverol Y. , Zorin A., Dass G., Vu M.T., Xu P., Glont M., Vizcaino J.A., Jarnuczak A.F., Petryszak R., Ping P. et al. Quantifying the impact of public omics data. Nat. Commun. 2019; 10:3512. 31383865 10.1038/s41467-019-11461-w PMC6683138

[2] Perez-Riverol Y. , Csordas A., Bai J., Bernal-Llinares M., Hewapathirana S., Kundu D.J., Inuganti A., Griss J., Mayer G., Eisenacher M. et al. The PRIDE database and related tools and resources in 2019: improving support for quantification data. Nucleic Acids Res. 2019; 47:D442–D450. 30395289 10.1093/nar/gky1106 PMC6323896

[3] Deutsch E.W. , Bandeira N., Sharma V., Perez-Riverol Y., Carver J.J., Kundu D.J., Garcia-Seisdedos D., Jarnuczak A.F., Hewapathirana S., Pullman B.S. et al. The ProteomeXchange consortium in 2020: enabling ‘big data’ approaches in proteomics. Nucleic Acids Res. 2020; 48:D1145–D1152. 31686107 10.1093/nar/gkz984 PMC7145525

[4] Ternent T. , Csordas A., Qi D., Gomez-Baena G., Beynon R.J., Jones A.R., Hermjakob H., Vizcaino J.A. How to submit MS proteomics data to ProteomeXchange via the PRIDE database. Proteomics. 2014; 14:2233–2241. 25047258 10.1002/pmic.201400120

[5] Griss J. , Jones A.R., Sachsenberg T., Walzer M., Gatto L., Hartler J., Thallinger G.G., Salek R.M., Steinbeck C., Neuhauser N. et al. The mzTab data exchange format: communicating mass-spectrometry-based proteomics and metabolomics experimental results to a wider audience. Mol. Cell. Proteomics. 2014; 13:2765–2775. 24980485 10.1074/mcp.O113.036681 PMC4189001

[6] Vizcaino J.A. , Mayer G., Perkins S., Barsnes H., Vaudel M., Perez-Riverol Y., Ternent T., Uszkoreit J., Eisenacher M., Fischer L. et al. The mzIdentML Data Standard Version 1.2, Supporting Advances in Proteome Informatics. Mol. Cell. Proteomics. 2017; 16:1275–1285. 28515314 10.1074/mcp.M117.068429 PMC5500760

[7] Martens L. , Chambers M., Sturm M., Kessner D., Levander F., Shofstahl J., Tang W.H., Rompp A., Neumann S., Pizarro A.D. et al. mzML–a community standard for mass spectrometry data. Mol. Cell. Proteomics. 2011; 10:R110 000133. 10.1074/mcp.R110.000133 PMC3013463 20716697

[8] Vizcaino J.A. , Deutsch E.W., Wang R., Csordas A., Reisinger F., Rios D., Dianes J.A., Sun Z., Farrah T., Bandeira N. et al. ProteomeXchange provides globally coordinated proteomics data submission and dissemination. Nat. Biotechnol. 2014; 32:223–226. 24727771 10.1038/nbt.2839 PMC3986813

[9] Perez-Riverol Y. , Xu Q.W., Wang R., Uszkoreit J., Griss J., Sanchez A., Reisinger F., Csordas A., Ternent T., Del-Toro N. et al. PRIDE Inspector Toolsuite: moving toward a universal visualization tool for proteomics data standard formats and quality assessment of ProteomeXchange datasets. Mol. Cell. Proteomics. 2016; 15:305–317. 26545397 10.1074/mcp.O115.050229 PMC4762524

[10] Yates A.D. , Achuthan P., Akanni W., Allen J., Allen J., Alvarez-Jarreta J., Amode M.R., Armean I.M., Azov A.G., Bennett R. et al. Ensembl 2020. Nucleic Acids Res. 2020; 48:D682–D688. 31691826 10.1093/nar/gkz966 PMC7145704

[11] UniProt C. UniProt: the universal protein knowledgebase in 2021. Nucleic Acids Res. 2021; 49:D480–D489. 33237286 10.1093/nar/gkaa1100 PMC7778908

[12] Papatheodorou I. , Moreno P., Manning J., Fuentes A.M., George N., Fexova S., Fonseca N.A., Fullgrabe A., Green M., Huang N. et al. Expression Atlas update: from tissues to single cells. Nucleic Acids Res. 2020; 48:D77–D83. 31665515 10.1093/nar/gkz947 PMC7145605

[13] Deutsch E.W. , Lam H., Aebersold R. PeptideAtlas: a resource for target selection for emerging targeted proteomics workflows. EMBO Rep. 2008; 9:429–434. 18451766 10.1038/embor.2008.56 PMC2373374

[14] Farrah T. , Deutsch E.W., Kreisberg R., Sun Z., Campbell D.S., Mendoza L., Kusebauch U., Brusniak M.Y., Huttenhain R., Schiess R. et al. PASSEL: the PeptideAtlas SRMexperiment library. Proteomics. 2012; 12:1170–1175. 22318887 10.1002/pmic.201100515 PMC3832291

[15] Choi M. , Carver J., Chiva C., Tzouros M., Huang T., Tsai T.H., Pullman B., Bernhardt O.M., Huttenhain R., Teo G.C. et al. MassIVE.quant: a community resource of quantitative mass spectrometry-based proteomics datasets. Nat. Methods. 2020; 17:981–984. 32929271 10.1038/s41592-020-0955-0 PMC7541731

[16] Moriya Y. , Kawano S., Okuda S., Watanabe Y., Matsumoto M., Takami T., Kobayashi D., Yamanouchi Y., Araki N., Yoshizawa A.C. et al. The jPOST environment: an integrated proteomics data repository and database. Nucleic. Acids. Res. 2019; 47:D1218–D1224. 30295851 10.1093/nar/gky899 PMC6324006

[17] Ma J. , Chen T., Wu S., Yang C., Bai M., Shu K., Li K., Zhang G., Jin Z., He F. et al. iProX: an integrated proteome resource. Nucleic Acids Res. 2019; 47:D1211–D1217. 30252093 10.1093/nar/gky869 PMC6323926

[18] Sharma V. , Eckels J., Schilling B., Ludwig C., Jaffe J.D., MacCoss M.J., MacLean B. Panorama public: a public repository for quantitative data sets processed in skyline. Mol. Cell. Proteomics. 2018; 17:1239–1244. 29487113 10.1074/mcp.RA117.000543 PMC5986241

[19] Deutsch E.W. , Perez-Riverol Y., Carver J., Kawano S., Mendoza L., Van Den Bossche T., Gabriels R., Binz P.A., Pullman B., Sun Z. et al. Universal Spectrum Identifier for mass spectra. Nat. Methods. 2021; 18:768–770. 34183830 10.1038/s41592-021-01184-6 PMC8405201

[20] Drysdale R. , Cook C.E., Petryszak R., Baillie-Gerritsen V., Barlow M., Gasteiger E., Gruhl F., Haas J., Lanfear J., Lopez R. et al. The ELIXIR Core Data Resources: fundamental infrastructure for the life sciences. Bioinformatics. 2020; 36:2636–2642. 31950984 10.1093/bioinformatics/btz959 PMC7446027

[21] Xu Q.W. , Griss J., Wang R., Jones A.R., Hermjakob H., Vizcaino J.A. jmzTab: a java interface to the mzTab data standard. Proteomics. 2014; 14:1328–1332. 24659499 10.1002/pmic.201300560 PMC4230411

[22] Reisinger F. , Krishna R., Ghali F., Rios D., Hermjakob H., Vizcaino J.A., Jones A.R. jmzIdentML API: a Java interface to the mzIdentML standard for peptide and protein identification data. Proteomics. 2012; 12:790–794. 22539429 10.1002/pmic.201100577 PMC3933944

[23] Perez-Riverol Y. , Uszkoreit J., Sanchez A., Ternent T., Del Toro N., Hermjakob H., Vizcaino J.A., Wang R. ms-data-core-api: an open-source, metadata-oriented library for computational proteomics. Bioinformatics. 2015; 31:2903–2905. 25910694 10.1093/bioinformatics/btv250 PMC4547611

[24] Uszkoreit J. , Perez-Riverol Y., Eggers B., Marcus K., Eisenacher M. Protein inference using PIA workflows and PSI standard file formats. J. Proteome Res. 2019; 18:741–747. 30474983 10.1021/acs.jproteome.8b00723

[25] Uszkoreit J. , Maerkens A., Perez-Riverol Y., Meyer H.E., Marcus K., Stephan C., Kohlbacher O., Eisenacher M. PIA: an intuitive protein inference engine with a web-based user interface. J. Proteome Res. 2015; 14:2988–2997. 25938255 10.1021/acs.jproteome.5b00121

[26] Perkins D.N. , Pappin D.J., Creasy D.M., Cottrell J.S. Probability-based protein identification by searching sequence databases using mass spectrometry data. Electrophoresis. 1999; 20:3551–3567. 10612281 10.1002/(SICI)1522-2683(19991201)20:18<3551::AID-ELPS3551>3.0.CO;2-2

[27] Cox J. , Mann M. MaxQuant enables high peptide identification rates, individualized p.p.b.-range mass accuracies and proteome-wide protein quantification. Nat. Biotechnol. 2008; 26:1367–1372. 19029910 10.1038/nbt.1511

[28] Pfeuffer J. , Sachsenberg T., Alka O., Walzer M., Fillbrunn A., Nilse L., Schilling O., Reinert K., Kohlbacher O. OpenMS–a platform for reproducible analysis of mass spectrometry data. J. Biotechnol. 2017; 261:142–148. 28559010 10.1016/j.jbiotec.2017.05.016

[29] Sinitcyn P. , Hamzeiy H., Salinas Soto F., Itzhak D., McCarthy F., Wichmann C., Steger M., Ohmayer U., Distler U., Kaspar-Schoenefeld S. et al. MaxDIA enables library-based and library-free data-independent acquisition proteomics. Nat. Biotechnol. 2021; 10.1038/s41587-021-00968-7. PMC8668435 34239088

[30] Perez-Riverol Y. , Ternent T., Koch M., Barsnes H., Vrousgou O., Jupp S., Vizcaino J.A. OLS client and OLS dialog: open source tools to annotate public omics datasets. Proteomics. 2017; 17:1700244. 10.1002/pmic.201700244 PMC5707441 28792687

[31] Mischak H. , Apweiler R., Banks R.E., Conaway M., Coon J., Dominiczak A., Ehrich J.H., Fliser D., Girolami M., Hermjakob H. et al. Clinical proteomics: a need to define the field and to begin to set adequate standards. Proteomics Clin Appl. 2007; 1:148–156. 21136664 10.1002/prca.200600771

[32] Griss J. , Perez-Riverol Y., Hermjakob H., Vizcaino J.A. Identifying novel biomarkers through data mining-a realistic scenario?. Proteomics Clin. Appl. 2015; 9:437–443. 25347964 10.1002/prca.201400107 PMC4833187

[33] Perez-Riverol Y. European Bioinformatics Community for Mass, S. Toward a sample metadata standard in public proteomics repositories. J. Proteome Res. 2020; 19:3906–3909. 32786688 10.1021/acs.jproteome.0c00376 PMC7116434

[34] Dai C. , Fullgrabe A., Pfeuffer J., Solovyeva E.M., Deng J., Moreno P., Kamatchinathan S., Kundu D.J., George N., Fexova S. et al. A proteomics sample metadata representation for multiomics integration and big data analysis. Nat. Commun. 2021; 12:5854. 34615866 10.1038/s41467-021-26111-3 PMC8494749

[35] Rayner T.F. , Rocca-Serra P., Spellman P.T., Causton H.C., Farne A., Holloway E., Irizarry R.A., Liu J., Maier D.S., Miller M. et al. A simple spreadsheet-based, MIAME-supportive format for microarray data: MAGE-TAB. BMC Bioinformatics. 2006; 7:489. 17087822 10.1186/1471-2105-7-489 PMC1687205

[36] Gostev M. , Faulconbridge A., Brandizi M., Fernandez-Banet J., Sarkans U., Brazma A., Parkinson H. The BioSample Database (BioSD) at the European Bioinformatics Institute. Nucleic Acids Res. 2012; 40:D64–D70. 22096232 10.1093/nar/gkr937 PMC3245134

[37] Schmidt T. , Samaras P., Dorfer V., Panse C., Kockmann T., Bichmann L., van Puyvelde B., Perez-Riverol Y., Deutsch E.W., Kuster B. et al. Universal spectrum explorer: a standalone (web-)application for cross-resource spectrum comparison. J. Proteome Res. 2021; 20:3388–3394. 33970638 10.1021/acs.jproteome.1c00096

[38] Griss J. , Perez-Riverol Y., Lewis S., Tabb D.L., Dianes J.A., Del-Toro N., Rurik M., Walzer M.W., Kohlbacher O., Hermjakob H. et al. Recognizing millions of consistently unidentified spectra across hundreds of shotgun proteomics datasets. Nat. Methods. 2016; 13:651–656. 27493588 10.1038/nmeth.3902 PMC4968634

[39] Qin C. , Luo X., Deng C., Shu K., Zhu W., Griss J., Hermjakob H., Bai M., Perez-Riverol Y. Deep learning embedder method and tool for mass spectra similarity search. J. Proteomics. 2021; 232:104070. 33307250 10.1016/j.jprot.2020.104070 PMC7613299

[40] Bittremieux W. , Laukens K., Noble W.S., Dorrestein P.C. Large-scale tandem mass spectrum clustering using fast nearest neighbor searching. Rapid Commun. Mass Spectrom. 2021; e9153 10.1002/rcm.9153. 34169593 PMC8709870

[41] Cook C.E. , Stroe O., Cochrane G., Birney E., Apweiler R. The European Bioinformatics Institute in 2020: building a global infrastructure of interconnected data resources for the life sciences. Nucleic Acids Res. 2020; 48:D17–D23. 31701143 10.1093/nar/gkz1033 PMC6943058

[42] Harrison P.W. , Lopez R., Rahman N., Allen S.G., Aslam R., Buso N., Cummins C., Fathy Y., Felix E., Glont M. et al. The COVID-19 Data Portal: accelerating SARS-CoV-2 and COVID-19 research through rapid open access data sharing. Nucleic Acids Res. 2021; 49:W619–W623. 34048576 10.1093/nar/gkab417 PMC8218199

[43] Brunet M.A. , Lucier J.F., Levesque M., Leblanc S., Jacques J.F., Al-Saedi H.R.H., Guilloy N., Grenier F., Avino M., Fournier I. et al. OpenProt 2021: deeper functional annotation of the coding potential of eukaryotic genomes. Nucleic Acids Res. 2021; 49:D380–D388. 33179748 10.1093/nar/gkaa1036 PMC7779043

[44] Shao X. , Taha I.N., Clauser K.R., Gao Y.T., Naba A. MatrisomeDB: the ECM-protein knowledge database. Nucleic Acids Res. 2020; 48:D1136–D1144. 31586405 10.1093/nar/gkz849 PMC6943062

[45] Ramasamy P. , Turan D., Tichshenko N., Hulstaert N., Vandermarliere E., Vranken W., Martens L. Scop3P: a comprehensive resource of human phosphosites within their full context. J. Proteome Res. 2020; 19:3478–3486. 32508104 10.1021/acs.jproteome.0c00306

[46] Kustatscher G. , Grabowski P., Schrader T.A., Passmore J.B., Schrader M., Rappsilber J. Co-regulation map of the human proteome enables identification of protein functions. Nat. Biotechnol. 2019; 37:1361–1371. 31690884 10.1038/s41587-019-0298-5 PMC6901355

[47] Omenn G.S. , Lane L., Overall C.M., Cristea I.M., Corrales F.J., Lindskog C., Paik Y.K., Van Eyk J.E., Liu S., Pennington S.R. et al. Research on the human proteome reaches a major milestone: >90% of predicted human proteins now credibly detected, according to the HUPO human proteome project. J. Proteome Res. 2020; 19:4735–4746. 32931287 10.1021/acs.jproteome.0c00485 PMC7718309

[48] Mitchell A.L. , Almeida A., Beracochea M., Boland M., Burgin J., Cochrane G., Crusoe M.R., Kale V., Potter S.C., Richardson L.J. et al. MGnify: the microbiome analysis resource in 2020. Nucleic Acids Res. 2020; 48:D570–D578. 31696235 10.1093/nar/gkz1035 PMC7145632

[49] Umer H.M. , Zhu Y., Pfeuffer J., Sachsenberg T., Lehtiö J., Branca R., Perez-Riverol Y. Generation of ENSEMBL-based proteogenomics databases boosts the identification of non-canonical peptides. 2021; bioRxiv doi:09 June 2021, preprint: not peer reviewed 10.1101/2021.06.08.447496. PMC8825679 34904638

[50] Watkins X. , Garcia L.J., Pundir S., Martin M.J., UniProt C. ProtVista: visualization of protein sequence annotations. Bioinformatics. 2017; 33:2040–2041. 28334231 10.1093/bioinformatics/btx120 PMC5963392

[51] Ochoa D. , Jarnuczak A.F., Vieitez C., Gehre M., Soucheray M., Mateus A., Kleefeldt A.A., Hill A., Garcia-Alonso L., Stein F. et al. The functional landscape of the human phosphoproteome. Nat. Biotechnol. 2020; 38:365–373. 31819260 10.1038/s41587-019-0344-3 PMC7100915

[52] Jarnuczak A.F. , Najgebauer H., Barzine M., Kundu D.J., Ghavidel F., Perez-Riverol Y., Papatheodorou I., Brazma A., Vizcaino J.A. An integrated landscape of protein expression in human cancer. Sci Data. 2021; 8:115. 33893311 10.1038/s41597-021-00890-2 PMC8065022

[53] Walzer M. , García-Seisdedos D., Prakash A., Brack P., Crowther P., Graham R.L., George N., Mohammed S., Moreno P., Papathedourou I. et al. Implementing the re-use of public DIA proteomics datasets: from the PRIDE database to Expression Atlas. 2021; bioRxiv doi:09 June 2021, preprint: not peer reviewed 10.1101/2021.06.08.447493. PMC9197839 35701420

[54] Bandeira N. , Deutsch E.W., Kohlbacher O., Martens L., Vizcaino J.A. Data management of sensitive human proteomics data: current practices, recommendations, and perspectives for the future. Mol. Cell. Proteomics. 2021; 20:100071. 33711481 10.1016/j.mcpro.2021.100071 PMC8056256

[55] Keane T.M. , O’Donovan C., Vizcaíno J.A. The growing need for controlled data access models in clinical proteomics and metabolomics. Nat. Commun. 2021; 12:5787. 34599180 10.1038/s41467-021-26110-4 PMC8486822

[56] Leitner A. , Bonvin A., Borchers C.H., Chalkley R.J., Chamot-Rooke J., Combe C.W., Cox J., Dong M.Q., Fischer L., Gotze M. et al. Toward increased reliability, transparency, and accessibility in cross-linking mass spectrometry. Structure. 2020; 28:1259–1268. 33065067 10.1016/j.str.2020.09.011

[57] Bai J. , Bandla C., Guo J., Vera Alvarez R., Bai M., Vizcaino J.A., Moreno P., Gruning B., Sallou O., Perez-Riverol Y. BioContainers Registry: searching bioinformatics and proteomics tools, packages, and containers. J. Proteome Res. 2021; 20:2056–2061. 33625229 10.1021/acs.jproteome.0c00904 PMC7611561

[58] Perez-Riverol Y. , Moreno P. Scalable data analysis in proteomics and metabolomics using BioContainers and workflows engines. Proteomics. 2020; 20:e1900147. 31657527 10.1002/pmic.201900147 PMC7613303

