# Supplementary material for: The PRIDE database resources in 2022: a hub for mass spectrometry-based proteomics evidences
Source: Nucleic Acids Res. 2021 Nov 1;50(D1):D543–52. doi: 10.1093/nar/gkab1038 (PMC8728295; doi:10.1093/nar/gkab1038)
Supplement: gkab1038_Supplemental_Files [file gkab1038_supplemental_files.zip › Supplementary Files.docx]

Supplementary Files

Supplementary File 1. Distribution of submitted datasets per species.
